# Berberine nanostructures attenuate ß-catenin, a key component of epithelial mesenchymal transition in lung adenocarcinoma

**DOI:** 10.1007/s00210-023-02553-y

**Published:** 2023-06-02

**Authors:** Vamshikrishna Malyla, Gabriele De Rubis, Keshav Raj Paudel, Dinesh Kumar Chellappan, Nicole G. Hansbro, Philip M. Hansbro, Kamal Dua

**Affiliations:** 1 Discipline of Pharmacy, Graduate School of Health, University of Technology Sydney, Sydney, NSW 2007 Australia; 2 Centre for Inflammation, Centenary Institute, Sydney, Sydney, NSW 2050 Australia; 3 Australian Research Centre in Complementary and Integrative Medicine, Faculty of Health, University of Technology Sydney, Ultimo, NSW 2007 Australia; 4 Faculty of Science, University of Technology Sydney, Sydney, NSW 2007 Australia; 5 Department of Life Sciences, School of Pharmacy, International Medical University, Bukit Jalil 57000, Kuala Lumpur, Malaysia

**Keywords:** Berberine, Nano-formulations, WNT/ß-catenin signalling pathway, Molecular simulation, Lung cancer

## Abstract

Lung cancer (LC) is the leading cause of cancer-related deaths globally. It accounts for more than 1.9 million cases each year due to its complex and poorly understood molecular mechanisms that result in unregulated cell proliferation and metastasis. β-Catenin is a developmentally active protein that controls cell proliferation, metastasis, polarity and cell fate during homeostasis and aids in cancer progression via epithelial–mesenchymal transition. Therefore, inhibition of the β-catenin pathway could attenuate the progression of LC. Berberine, an isoquinoline alkaloid which is known for its anti-cancer and anti-inflammatory properties, demonstrates poor solubility and bioavailability. In our study, we have encapsulated berberine into liquid crystalline nanoparticles to improve its physiochemical functions and studied if these nanoparticles target the β-catenin pathway to inhibit the human lung adenocarcinoma cell line (A549) at both gene and protein levels. We observed for the first time that berberine liquid crystalline nanoparticles at 5 µM significantly attenuate the expression of the β-catenin gene and protein. The interaction between berberine and β-catenin was further validated by molecular simulation studies. Targeting β-catenin with berberine nanoparticles represents a promising strategy for the management of lung cancer progression.

**Supplementary information:**

The online version contains supplementary material available at 10.1007/s00210-023-02553-y.

## Introduction

Lung cancer (LC) is the leading cause of cancer-related deaths globally, accounting for more than 1.9 million cases each year. This is due to a lack of early diagnostic tools and effective pharmacological interventions, as well as high prevalence rates of cigarette smoking. Understanding the pathology of LC is vital for developing novel diagnostic and therapeutic interventions (Siegel, et al. 2022; Solanki et al. 2020). One of the primary reasons for the increasing number of lung cancer cases is the lack of effective diagnostic and therapeutic approaches. Understanding the molecular mechanisms responsible for lung cancer pathogenesis, and improving strategies to inhibit specific pathways at the molecular level, could aid in controlling tumorigenesis and metastasis (Malyla et al. 2020). Current approaches for the management of LC include chemotherapy, immunotherapy, targeted therapy, radiation therapy and surgical removal of lung tumour (Paudel et al. 2020; Wadhwa, et al. 2020). With regard to chemotherapy, the major drawbacks of the currently available synthetic drug molecules are poor bioavailability and off-target effects (Cui et al. 2018). Alternatively, there is a growing interest in natural product-derived phytoconstituents, which represent a promising option for the therapeutic targeting of LC. Berberine is an isoquinoline alkaloid abundantly found in the stems, barks and roots of plants belonging to the genus *Berberis*, which is widely known for its anti-cancer and anti-inflammatory properties (Li et al. 2014; Mehta et al. 2021). Berberine has shown to inhibit non-small cell lung cancer (NSCLC) growth through repression of DNA repair and replication (Ni et al. 2022) and through induction of apoptosis (Chen et al. 2022; Chen et al. 2019; Li et al. 2018), as well as through inhibition of proliferation and colony formation capacity (Chen et al. 2020). Furthermore, berberine prevents LC by inhibiting EMT-promoting macrophages overexpressing the protein arginine deaminase 4 (PADI4) (Gu et al. 2022), and it reduces LC cell expression of PD-L1, thus facilitating antitumor immune response (Liu et al. 2020). Despite the promising anti-cancer activity and enormous potential, the major drawbacks of berberine, and many other plant-derived active principles, are low bioavailability and limited bio-absorption, which limit their in vivo efficacy and therapeutic use (Battu et al. 2010). A viable strategy to overcome these drawbacks, particularly for lung and respiratory diseases that allow the administration of drugs through inhalation, is represented by the encapsulation of active principles in advanced nanoparticles/nanocarrier systems (Doroudian et al. 2019). Liquid crystalline nanoparticles (LCNPs), among many emerging nanoparticle-based delivery systems, have been gaining momentum as nanocarrier systems for antioxidant and anti-cancer drugs as they increase the drug’s stability, bio-adhesiveness, sustained release properties, bioavailability, drug loading capacity and physicochemical stability (Paudel, et al. 2022). Recent studies have demonstrated the potent in vitro anti-cancer activity of berberine-loaded LCNPs, with an inhibitory effect on the proliferation, colony formation, migration and invasion capacity of NSCLC cells (Mehta et al. 2021; Alnuqaydan, et al. 2022; Paudel et al. 2022). Mechanistically, these effects are mediated by the inhibition of the expression of EMT-related proteins (Snail, P27 and vimentin) and migration and proliferation-related proteins such as BCLx, galectin-3 and survivin (Paudel et al. 2022), as well as by the upregulation of the mRNA levels of tumour suppressors PTEN and p53 downregulation of the oncogene KRT18 (Alnuqaydan, et al. 2022). Furthermore, berberine LCNPs downregulate the expression of inflammation/oxidative stress-related cytokines such as CCL20, CXCL-8 and HO-1 (Mehta et al. 2021).

Although berberine-loaded LCNPs represent a valuable option in the treatment of LC, further research is still needed to completely characterise the pathways that are affected by this treatment which results in such a promising anti-cancer activity. WNT/β-catenin is a developmentally active signal transduction pathway that controls cell proliferation, metastasis, polarity and cell fate during homeostasis (Zhang and Wang 2020). In a normal state, the β-catenin destruction complex generally recruits dishevelled (DVL), a scaffolding protein which activates the Axin complex (glycogen synthase kinase 3 (GSK3), casein kinase 1 (CK1) and adenomatous polyposis coli (APC)). This complex attenuates β-catenin. However, during pathological states, WNT markers bind to a transmembrane frizzled receptor, destabilising the β-catenin destruction complex. This would further leads to the accumulation of β-catenin in the cytoplasm. The accumulated β-catenin translocates to the nucleus, resulting in the activation of various oncogenic pathways that regulate the transcription factors in the T cell factor/lymphoid enhancer factor family (TCF/LEF) as well as the epithelial–mesenchymal transition (EMT)-related genes (Fig. 3) (Anthony et al. 2020; Cadigan and Waterman 2012).β-Catenin has a multifaceted role. It is a component of the cadherin-based cell–cell communication system, which helps in maintaining polarity and regulating tight junctions (Meng and Takeichi 2009). In cancer, polarised epithelial cells change their morphology to a mesenchymal phenotype through a process called EMT (Thiery, et al. 2009), which is a key process in embryogenesis and cancer. This process is controlled by β-catenin through regulation of EMT molecules such as cadherins (Heuberger and Birchmeier 2010), Twist family BHLH transcription factor (Twist) (Li and Zhou 2011), Snail1, slug (Stemmer, V.d,, et al. 2008), and zinc finger E-box binding homeobox 1 (ZEB1) (Sánchez-Tilló et al. 2011). Therefore, β-catenin is a key agent in cancer progression and EMT, and inhibition of this system could suppress the pathogenesis and attenuate the progression of LC (Valenta et al. 2012).

In this study, we show that treatment of human adenocarcinoma cells (A549) with berberine LCNPs significantly reduced the expression of WNT/β-catenin pathway markers β-catenin and Axin1 at a low concentration of 5 μM berberine. Furthermore, we have identified, through docking studies and molecular dynamics simulations, a putative binding site for berberine on β-catenin. This study provides further proof of the improved solubility and stability of berberine which is encapsulated in LCNs as a novel therapeutic approach against LC, shedding light on a previously unexplored mechanism by which berberine-loaded LCNs exerted anti-cancer activity.

## Materials

Berberine hydrochloride, monoolein (MO) and poloxamer 407 (P407) were purchased from Sigma Chemical Co, Germany. Spectra/Por dialysis membrane bags (dialysis tubing 238 cellulose membrane; molecular-weight-cut-off: 14,000 g/mol) were purchased from Sigma-Aldrich, USA. Culture media and reagents were of analytical and molecular grade and were used without any further purification.

## Methods

### Cell culture

A549 adenocarcinoma human alveolar basal epithelial cell line (ATCC, USA) was obtained as a gift from Prof. Alaina Ammit, Woolcock Institute of Medical Research, Sydney, Australia. The cells were grown in a RPMI media (Sigma-Aldrich, USA) containing 5% foetal bovine serum (Novogen, Australia), 1% penicillin and streptomycin (Gibco, New York) and were maintained at 37 °C in an incubator with 5% CO_2_. Cells were constantly checked for mycoplasma contamination, and all experiments were conducted in mycoplasma-negative cells.

### Preparation of berberine-loaded liquid crystal nanoparticles (LCNPs)

Berberine-loaded liquid crystalline nanoparticles were formulated as previously reported, using the ultrasonication technique (Paudel 2022). Briefly, 5 mg berberine was completely dissolved in 200 mg melted MO at 70 °C. This berberine–MO mixture was then mixed with the surfactant solution (20 mg P407 dissolved in 4.8 mL water), obtaining a coarse dispersion. This dispersion was size-reduced using a probe sonicator (Labsonic® P, Sartorius, Germany), with the amplitude maintained at 80, and 5-s on and 5-s off-cycles for 5 min. This resulted in the production of 1 mg/mL berberine-loaded MO-LCNPs, with a lipid content of 40 mg/mL and a lipid:surfactant ratio of 1:10 w/w (Wadhwa et al. 2021).

### Cytotoxicity (cell proliferation) assay of berberine LCNPs (BBR LNCPs) in A549 cells

A549 cells were plated in a 6-well plate at a density of 2 × 10^5^ cells/well. Cells were treated with or without 5 μM of BBR LCNPs for 24 h. The dose of 5 μM of BBR LCNPs was decided from our previously published study showing significant anti-proliferative (MTT assay) and anti-migratory activity of berberine against the A549 cell line at that concentration (Paudel 2022).

### RNA extraction and assessment of gene expression level by real-time PCR

RNA was extracted using the TRIzol® method. Extracted RNA was reverse transcribed to cDNA using a high-capacity DNA reverse transcription kit (Applied Biosystems, RI, USA). Real-time PCR analysis was performed using TaqMan primers and probes (Applied Biosystems) to measure the relative expression of genes CTTNB1 (forward primer: CTTGGAATGAGACTGCTG; reverse primer: AGAGTGAAAAGAACGATAGC), Axin1 (forward primer: CCGACCTTANATGAAGATGAG; reverse primer: CAGGATCCATACCTGAACTC). The quantitative expression of these genes was calculated through the 2∧-Ct method and was compared to the respective reference gene (GAPDH). The relative transcript abundance in the treated groups was calculated by comparing the treated groups with untreated controls (Wadhwa et al. 2021).

### Immunofluorescence (IF)

Coverslips were sterilised and coated with placenta collagen (Sigma cat. no. C5533) for 30 min and washed twice with 1 ml 1 × PBS to allow cell adhesion. Then, 2 × 10^5^ A549 cells were seeded on the cover slip and placed in a 6-well plate. The following day, cells were treated with 5 μM BBR LCNPs for 24 h. The media was then removed, and cells were washed with sterile 1 × PBS. Cells were fixed with ice-cold methanol for 20 min, followed by blocking with 2% bovine serum albumin (BSA) for 1 h at room temperature. The cells were then incubated with anti-beta catenin (ab32572) antibody at 1:250 dilution overnight at 4 °C, followed by incubation with goat anti-rabbit IgG H&L Alexa Fluor® 647 (ab150083) for 1 h at room temperature (1:500 dilution), protected from light. Finally, the slides were cover slipped with DAPI fluoromount G (ProSciTech). The microscopic images at random fields were then captured with a fluorescence microscope (Zeiss microscope, Germany) at 40 × magnification (Paudel and Kim 2020).

### Protein extraction and quantification

RIPA buffer (Sigma-Aldrich) containing phosphatase and protease inhibitor cocktail (Roche, USA) was added to the 6-well plates containing treated and untreated cells, and the lysate was collected in a microcentrifuge tube. Proteins were extracted from lysed cells upon vortex treatment and incubation on ice for 15 min. Soon after, the mixture was sonicated at 30% amplitude, 3 times for 2 s. The mixture was then incubated again on ice for an additional 15 min. The lysate was then centrifuged for 30 min at 18,000 g at 4 °C. Finally, the supernatant was collected, and the proteins were quantified using a PierceTM BCA Protein Array Kit (Thermo Scientific) following the manufacturer’s instructions (Wadhwa et al. 2021).

### Immunoblotting

Equal amounts of the extracted proteins were loaded on SDS-PAGE gels and transferred to a PVDF membrane. The membrane was blocked with 5% BSA and incubated with primary rabbit anti-beta catenin antibody (ab 32,572) at a 1:7500 dilution overnight at 4 °C, followed by incubation with goat anti-rabbit IgG at a 1:10,000 dilution for 1 h. Images were acquired using a chemiDoc imaging system. Then, the membrane was stripped and blocked again with 5% BSA. It was then incubated with mouse anti-beta actin antibody (ab8226) at 1:10,000 dilution at room temperature for 3 h followed. After this, the membrane was incubated with anti-mouse IgG at 1:10,000 dilution, and images were again acquired using a chemiDoc imaging system (whole blots were added in the supplementary file). The densitometric analysis of proteins was performed by quantifying pixel density using ImageJ software.

### Statistical analysis

The values are represented as mean ± SEM. GraphPad Prism (version 9.3) was used to perform statistical analyses. Statistical comparisons were done by unpaired, two-tailed student’s *t*-test. A value of *p* < 0.05 was considered statistically significant.

### Molecular docking study

For the molecular docking study, the three-dimensional structure of human beta catenin (PDB ID: 1JDH) and berberine (PubChem CID 2353) was retrieved from the Protein Data Bank (PDB) and PubChem, respectively (Graham et al. 2001; Berman et al. 2000; Kim et al. 2016). Before molecular docking, the protein structure was prepared by adding hydrogens, optimising geometry and minimising energy with the protein preparation wizard of Schrodinger (Sastry et al. 2013). Similarly, the ligand geometry was optimised with the LigPrep module of Schrodinger (Sastry et al. 2013). The molecular docking study was performed using Vina wizard of PyRx software (Dallakyan and Olson 2015). The grid was generated around the β-catenin residues Phe253, His260, Asn261, Leu264, Asn290, Lys292, Phe293, Ile296, Asp299, Gln302, Tyr306, Gly307, Lys312, Lys335, Arg342, Lys345, Val346, Arg376, Arg386, Asn387, Asp390, Gly422, Ser425, Asn426, Cys429, Asn430, Lys435, Arg469, His470, Ser473, Arg474, Lys508, Gly512, Asn516 and Leu519. These residues were selected because they were reported to be involved in the interaction between β-catenin and human TCF4, in a study in which the β-catenin–hTcf4 complex was characterised through X-Ray crystallography (Graham et al. 2001). The coordinates of *X*, *Y* and *Z* were set to 73.8, − 19.75 and − 15.46, respectively. The exhaustiveness of the algorithm is set to 8. After molecular docking, the top docking pose of the ligand was selected based on the docking score and subjected to molecular dynamics simulation.

### Molecular dynamics study

The molecular dynamics (MD) simulation was run using the Desmond software (Bowers, et al. 2006). For MD simulation, the system was prepared by adding water and neutralised by the addition of sodium and chloride ions. The berberine–β-catenin complex was hydrated in an orthorhombic box using a three-point water model (TIP3P) (Bowers, et al. 2006). The implicit solvent system was used to represent solvent. After that, the system was energy minimised. Finally, the minimised system was subjected to 50 ns of MD simulations in isothermal–isobaric ensemble (NPT) at high temperature. Fifty ns of MD simulations were deemed sufficient for the MD simulation, considering the high-temperature settings, the use of the implicit solvent system and the fact that the backbone chains of carbon atoms showed stability throughout the 50 ns. The final trajectory was analysed with manual inputs and root mean square deviation (RMSD), protein–ligand contact histogram, protein–ligand contact diagram and root mean square fluctuation (RMSF) calculated using the simulation interaction diagram utility of maestro.

### MMGBSA energy calculation

After MD simulation, to estimate the binding affinity of the berberine–β-catenin complex, the binding-free energy (dG_Bind) was estimated using the thermal_mmgbsa.py script of Schrodinger (Genheden and Ryde 2015). The calculation was performed on 100 complexes obtained by extracting every 10th frame from the last 10 ns of the stable trajectory. After MMGBSA calculation, an average binding-free energy was reported.

## Results

**Fig. 1:**
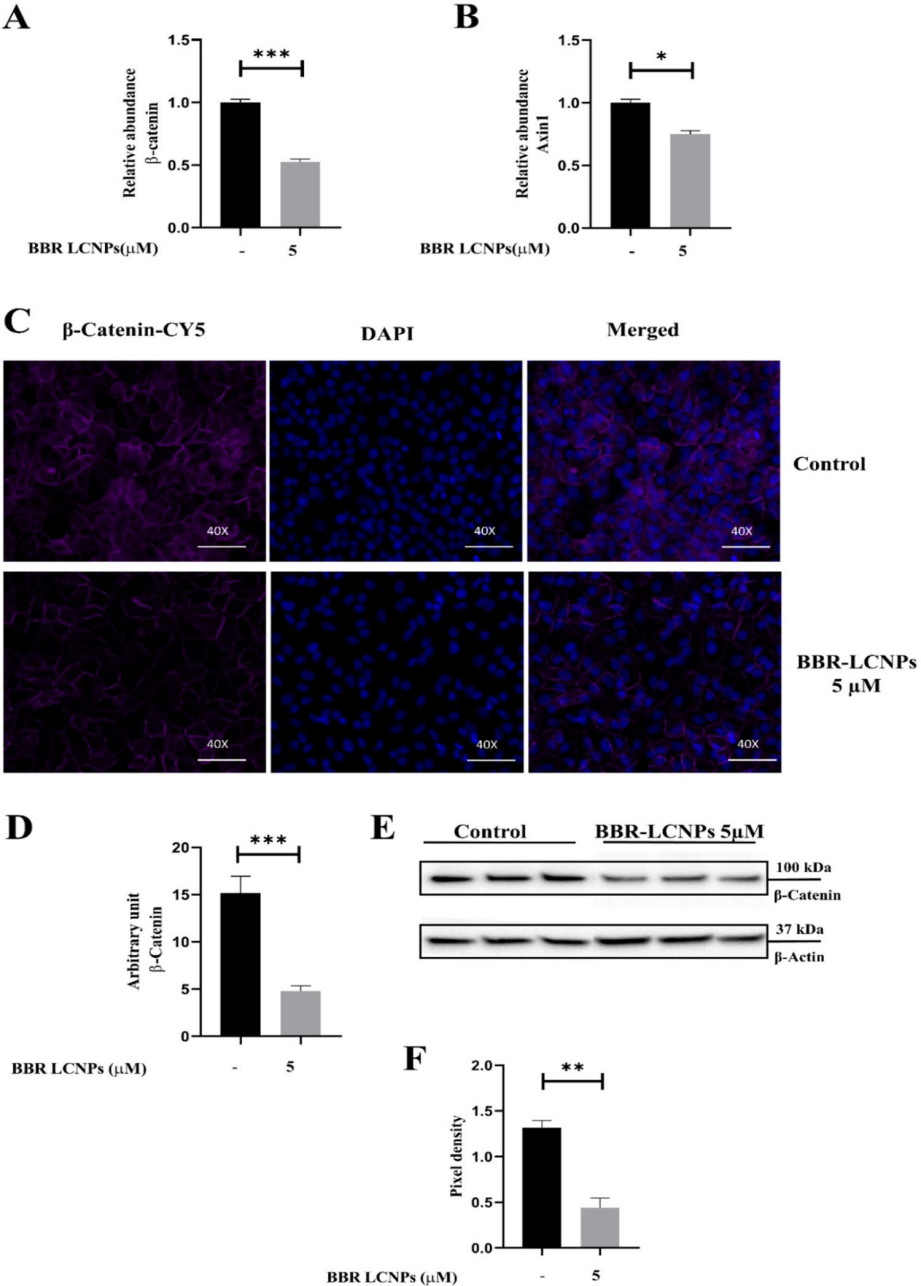
Effect of BBR LCNPs in the reduction of WNT/β-catenin pathway genes β-catenin and Axin1. After 24-h treatment of A549 cells with BBR LCNPs, the expression of WNT/β-catenin pathway genes was assessed via RT-PCR, IF and immunoblotting. **A** β-Catenin expression, RT-PCR. **B** Axin1 expression, RT-PCR. The values are represented as mean ± SEM of 3 independent experiments, *p < 0.05, ***p < 0.001. **C** Immunofluorescence staining for β-catenin under 40 × magnification. **D** Fluorescence quantification with ImageJ Fiji. The values represent mean ± SEM of 3 independent experiments, ***p < 0.001. **E** β-Catenin expression, immunoblotting. **F** Pixel density quantification of immunoblotting with ImageJ Fiji. The values represent mean ± SEM of 3 independent experiments, ** *p* ≤ 0.01

Based on the cytotoxicity studies reported in our previously published work on the effect of BBR LCNPs on A549 cells, we identified 5 μM as the optimal concentration for anti-cancer activity of the formulation. This is validated by anti-proliferative, anti-migratory and colony formation assays (Wadhwa et al. 2021). Based on these data, we proceeded with 5 μM BBR LCNPs as an optimal concentration for studying the effect of these NPs on β-catenin activity. To begin with, we initially validated the activity of the formulation at the transcript level using RT-PCR. In this experiment, a 24-h treatment of A549 cells with BBR LCNPs at 5 μM resulted in a significant downregulation of β-catenin (Fig. 1A) and Axin1 (Fig. 1B), the key components of the WNT/β-catenin pathway, as compared to untreated control (only A549 cells). BBR LCNs decreased β-catenin expression to 0.5-fold and Axin1 expression to approximately 0.7-fold. This made us to look the activity at the protein level, and we performed an immunofluorescence assay to quantify β-catenin protein expression. As shown in Fig. 1C, a 24-h treatment of A549 cells with BBR LCNPs at 5 μM resulted in a significant decrease in the intensity of the β-catenin fluorescence signal. The quantification of fluorescence intensity showed that BBR LCNP treatment decreased the β-catenin intensity by tenfold (Fig. 1D). A similar trend was observed with western blotting (Fig. 1E). Overall, BBR LCNPs had reduced the expression of β-catenin at both transcript and protein levels. To further understand the putative region where BBR binds to β-catenin, we performed molecular docking and molecular dynamic simulation studies.

**Fig. 2:**
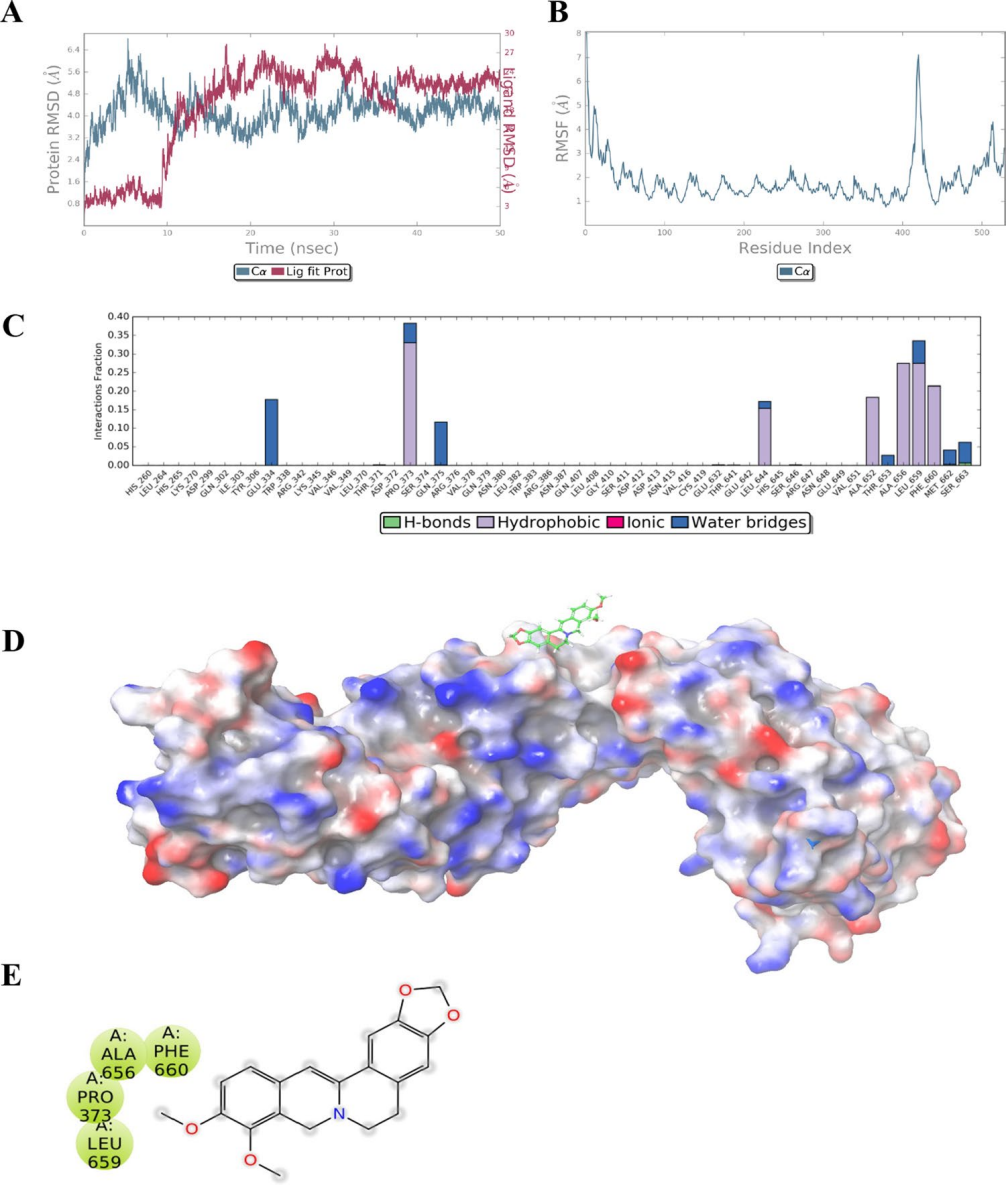
Molecular docking and simulation studies with berberine and β-catenin. **A** Root mean square deviation (RMSD) indicates the stability of β-catenin with berberine. **B** Root mean square fluctuation (RMSF) plot of berberine–β-catenin complex. **C** Berberine–β-catenin contact summary calculated from the last 10 ns of the stable trajectory. **D** Surface representation of the HTCF-4 binding site of berberine with β-catenin. **E** Two-dimensional representation of the residues of β-catenin surrounding berberine

The molecular docking study was performed to explore the best binding pose of berberine with β-catenin. Among all the generated poses of berberine, the best pose exhibited a binding energy of − 6.4 kcal/mol in the human TCF4 binding site. To assess the stability of berberine in its predicted binding site, a molecular dynamics simulation was performed. Molecular dynamics simulations represent a robust tool to understand the behaviour of protein–ligand complexes in dynamic conditions. As observed from the RMSD plot (Fig. 2A), β-catenin remained stable throughout the 50-ns simulation (root mean square deviation 4.17 Å). The maximum RMSF was shown by residues 543–558 of β-catenin (Fig. 2B). The ligand berberine remained stable in the human T cell transcription factor-4 (HTCF-4) binding site for the first 10 ns of simulation time, after which it left the binding site and occupied a site far away from the HTCF-4 binding site, where it remained stable for the remainder of the simulation period (Fig. 2D). At its binding site, berberine remained stable by interacting majorly with hydrophobic resides Pro373, Leu644, Ala652, Ala656, Leu659 and Phe660 via hydrophobic interactions and to a lesser extent with Glu334, Gln375, Thr653, Met662 and Ser663 via water bridges (Fig. 2C). Moreover, berberine exhibited an average free energy of binding (dG_Bind) of − 26.82 kcal/mol (Table 1). Berberine was observed binding to β-catenin predominantly via lipophilic (− 16.52 kcal/mol) and van der Waals (− 17.41 kcal/mol) interactions.

**Table 1:** The energy components of free energy of binding of berberine–β-catenin complex calculated with MMGBSA programme

Average dG_Bind	Average dG_Bind_Coulomb	Average dG_Bind_Lipophilic	Average dG_Bind_vdW	Average dG_Bind_Hbond
− 26.82	− 2.46	− 16.52	− 17.41	− 0.11

## Discussion

Although berberine possesses several therapeutic activities, it is a very hydrophobic molecule with a poor solubility of ∼2.0 mg/mL resulting in low oral bioavailability (Battu et al. 2010). To achieve potent biological activity, berberine should be administered in high doses. However, administration of high doses might not be favourable due to its toxicity in healthy/normal cells. In a published study, Wu Ke et al. have shown that treatment with pure berberine powder at 10 μM concentration in DMSO resulted in significant inhibition of the WNT/β-catenin pathway in HCT116 colon cancer cells (Wu et al. 2012). To overcome these drawbacks of poor solubility and improved activity at lower concentrations, in this study, we encapsulated BBR in LCNPs and found that at 5 μM (half dose compared to Wu Ke et al. was sufficient to inhibit WNT/β-catenin pathway’s key component β-catenin at both the transcript and protein levels). This demonstrates the advantage of employing berberine-encapsulated LCNPs rather than pure berberine powder for improved activity at lower doses. Furthermore, in a previous study, we have shown that the physicochemical parameters of our BBR LCNP formulation (in terms of particle size, polydispersity index, in vitro release and entrapment efficiency) were significantly favourable (Paudel 2022).

**Fig. 3:**
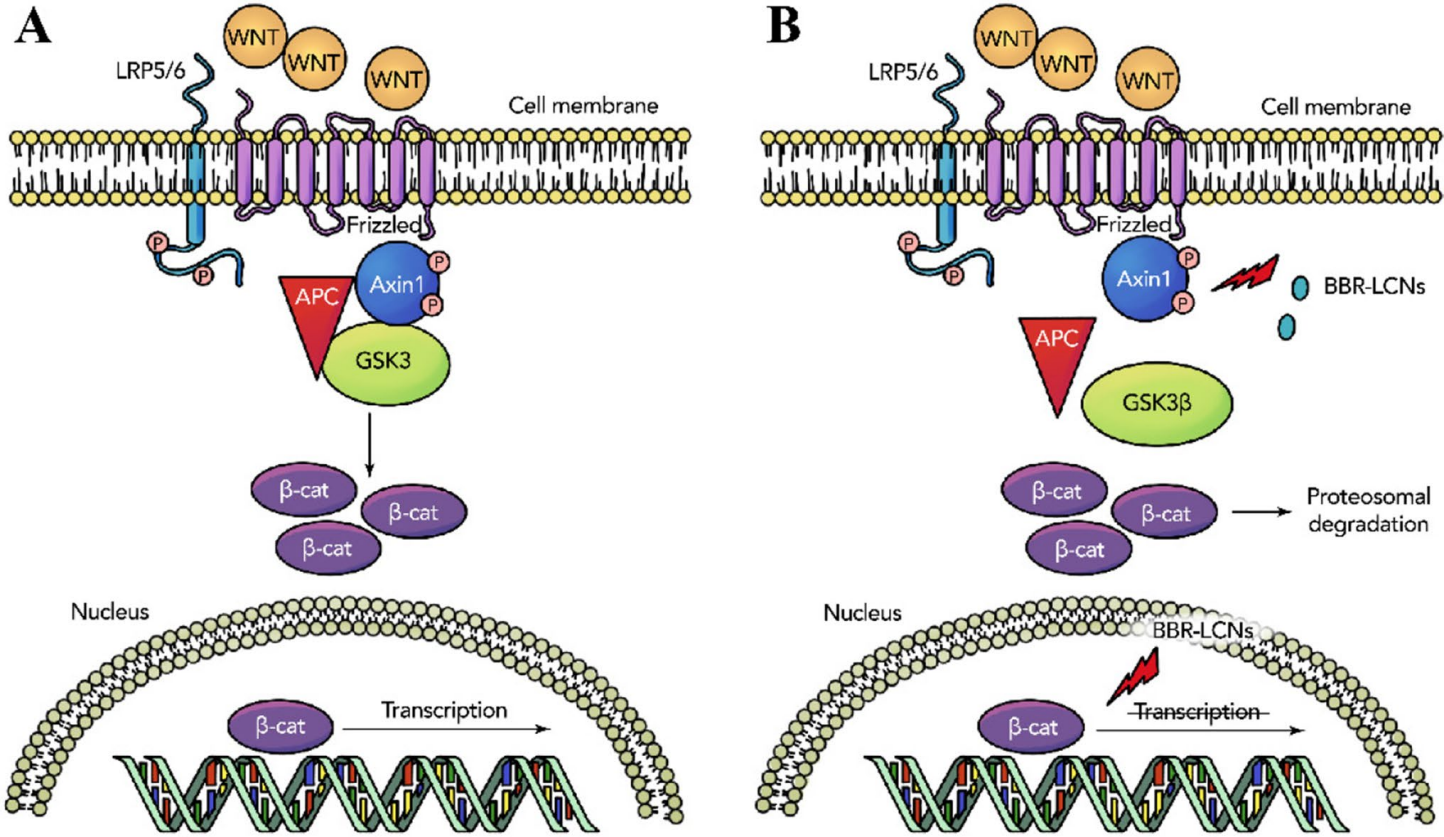
Predicted molecular mechanism of action of BBR LCNP treatment on A549 cells. **A** The normal state of WNT/β-catenin pathway is shown, where binding of WNT molecules leads to the activation of β-catenin resulting in tumorigenesis. **B** Inhibition of AXIN1 and β-catenin by BBR LCNPs is shown

The WNT/β-catenin pathway is a key activator of various genes related to EMT and cancer, including LC (Valenta et al. 2012; Stewart 2014). The inhibition of the key EMT regulator β-catenin can be crucial in understanding the progression of LC. Previously, we found a significant reduction of EMT markers like Snail, P27 and vimentin in LC. However, the effect of the formulation on β-catenin remains unknown (Paudel 2022). In the present study, treatment of BBR LCNPs to human lung adenocarcinoma cells (A549) significantly reduced the expression of the WNT/β-catenin pathway markers β-catenin and Axin1 at transcript level and β-catenin at protein level at a concentration of just 5 μM, which is two times lower than previously reported studies (Albring et al. 2013). The inhibition of Axin1, a key component of the APC destruction complex which regulates and stabilises β-catenin, represents a further way to inhibit the WNT/β-catenin pathway (Fig. 3A and B) (Jiang et al. 2018). This data provides proof that BBR LCNPs inhibit β-catenin via the canonical WNT/β-catenin pathway.

Although, among the chemicals used to formulate the LCNPs, no component was specifically reported to have anti-cancer activity, treatment of cancer cells with LCNPs would provide proof of any eventual contributing biological activity mediated by the LCNPs itself, further confirming that the anti-cancer action is mediated by the berberine itself. This will be assessed in future studies in vivo setting which currently are in progress in our laboratory.

Molecular docking studies suggest that berberine binds to β-catenin at the human TCF4 binding site and interacts with various amino acids via hydrophobic and water bridges with amino acids such as Phe660, which is critical for the binding of β-catenin to TCF inhibitors (Graham et al. 2002). Park et al. identified Ser663 as the amino acid where phosphorylation of β-catenin takes place. This event is vital for transcriptional regulation. As a result, the interaction of berberine with these residues may be critical for berberine’s β-catenin inhibitory effect (Park et al. 2012).

## Conclusions and future perspectives

In conclusion, natural product-derived phytochemicals can be a potential alternative for lung cancer treatment, as they assist to overcome the drawbacks that exist with synthetic anti-cancer drugs such as high cost, adverse reactions and unfavourable effects on the immune system. However, the use of natural phytochemicals such as berberine also has limitations such as poor solubility and low bioavailability. Therefore, the use of nanotechnology and advanced drug delivery approaches to design LCNPs loaded with berberine has improved its physiochemical properties resulting in improved efficacy. The potent anti-cancer activity of this innovative therapeutic tool, obtained through the suppression of β-catenin by BBR LCNPs at a low dose of 5 μM, at both protein and gene levels is highly promising and requires further mechanistic understanding. Molecular dynamics studies found that BBR binds at Ser663, which is required for phosphorylation of β-catenin. This indicates that BBR might inhibit the phosphorylation of β-catenin at protein level. Future directions of this research should include further characterisation of the detailed in vitro mechanistic effects, as well as the validation of BBR LCNPs in pre-clinical animal models of LC. Furthermore, the clinical application of this promising nanoparticle-based therapeutic approach would benefit from the assessment of its suitability as a pulmonary drug delivery system.

### Supplementary information

Below is the link to the electronic supplementary material.

Supplementary file1 (DOCX 4519 KB)

## Data Availability

All the data will be available upon request.

## Abbreviations

APC: Adenomatous polyposis coli
BBR: Berberine
BSA: Bovine serum albumin
CK1: Casein kinase 1
CTNNB1: Catenin beta 1
DAPI: 4′,6-Diamidino-2-phenylindole
DMSO: Dimethyl sulfoxide
DVL: Dishevelled
EMT: Epithelial–mesenchymal transition
GAPDH: Glyceraldehyde 3-phosphate dehydrogenase
GSK3: Glycogen synthase kinase 3
hTCF-4: Human T cell transcription factor-4
IF: Immunofluorescence
LC: Lung cancer
LCNP: Liquid crystalline nanoparticle
MD: Molecular dynamics
MMGBSA: Molecular mechanics with generalised born and surface area solvation
MO: Monoolein
MTT: 3-(4,5-Dimethylthiazol-2-yl)-2,5-diphenyltetrazolium bromide
P407: Poloxamer 407
PADI4: Protein arginine deaminase 4
PBS: Phosphate-buffered saline
RIPA buffer: Radioimmunoprecipitation assay buffer
RMSD: Root mean square deviation
RMSF: Root mean square fluctuation
SDS-PAGE: Sodium dodecyl sulphate–polyacrylamide gel electrophoresis
TCF/LEF: T cell factor/lymphoid enhancer factor family
TIP3P: Three-point water model
ZEB1: Zinc finger E-box binding homeobox 1
