# Supplementary material for: Berberine nanostructures attenuate ß-catenin, a key component of epithelial mesenchymal transition in lung adenocarcinoma
Source: Naunyn Schmiedebergs Arch Pharmacol. 2023 Jun 2;396(12):3595–603. doi: 10.1007/s00210-023-02553-y (PMC10643271; doi:10.1007/s00210-023-02553-y)

A549 BBR LCNs beta-catenin full blot

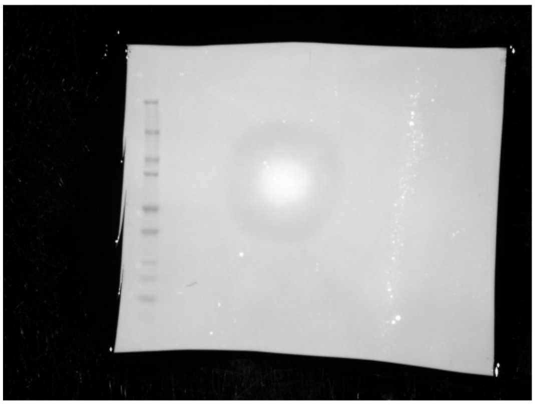

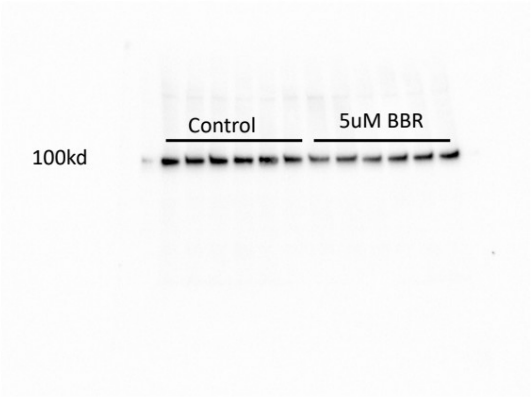

A549 BBR LCNs beta-Actin full blot

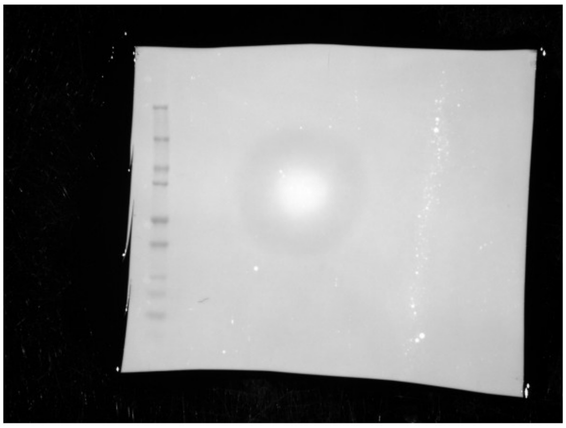

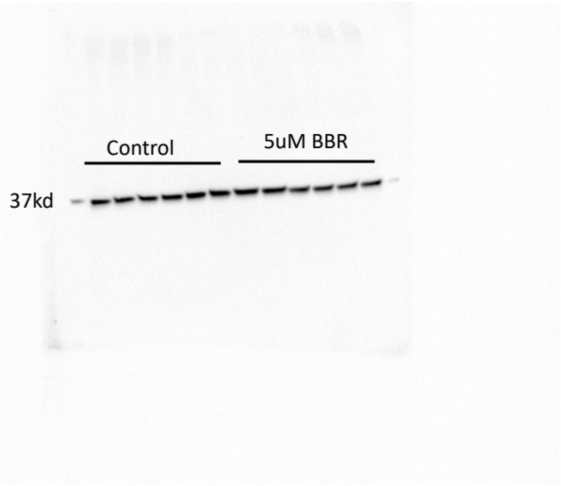

Supplement: Supplementary file 1 [file 210_2023_2553_MOESM1_ESM.docx]
